# Long-Term Knee Health in Adults with a History of Adolescent Osgood–Schlatter: A National Cohort Study of Patients in Secondary Care in Denmark 1977–2020

**DOI:** 10.1007/s40279-025-02214-5

**Published:** 2025-05-29

**Authors:** Kasper Krommes, Amalie Bjerre, Kristian Thorborg, Mathias Fabricius Nielsen, Per Hölmich

**Affiliations:** 1 Sports Orthopedic Research Center—Copenhagen, Orthopedic Department, Amager-Hvidovre, Copenhagen University Hospital, Copenhagen, Denmark; 2 Department of Clinical Medicine, University of Copenhagen, Copenhagen, Denmark; 3 Faculty of Health and Medical Sciences, University of Copenhagen, Copenhagen, Denmark

## Abstract

**Background:**

Osgood–Schlatter has, until recently, been suggested to be a benign condition, affecting adolescents in terms of knee pain and decreased sports participation during growth, with no long-term consequences seen later in adulthood.

**Objectives:**

The objectives of this study were to describe the long-term knee health in adults with a history of Osgood–Schlatter, compare these findings with healthy population estimates, and investigate if explanatory variables are associated with current knee health.

**Methods:**

The Danish Patient Registry identified patients ≥ 18 years diagnosed with adolescent Osgood–Schlatter in hospitals during 1977–2020. All cases participated in a survey about knee-related health and comorbidities. Existing literature was sourced for the healthy population estimates for comparisons. Explanatory variables were recalled Osgood–Schlatter duration, pain levels, restrictions, and current tibial tubercle prominence.

**Results:**

Of 1281 identified patients, 400 responded. Most reported having a current bony prominence of the tibial tubercle (85%) and sustained pain/problems from the same area (73%). Compared with healthy population estimates, Osgood–Schlatter cases scored lower on the Knee Injury and Osteoarthritis Outcome Score on all subscales (*p* < 0.05), particularly for “sport/rec” and “quality of life” (Cohen’s *d* > 0.8). Similarly, cases exhibited a large risk of “jumper’s knee” (odds ratio: 70.4 [95% confidence intervals, CI: 32.9; 155.0], *p* < 0.0001). Symptom duration and pain levels were negatively associated with several outcomes (*p* < 0.05).

**Conclusions:**

Adults with a history of Osgood–Schlatter have significantly worse long-term knee health than what is observed from healthy population estimates. Recalled longer symptom duration and higher pain levels were associated with worse current knee health. This information should potentially guide management to maintain knee health over time, as the condition is not always as benign and self-limiting as previously thought.

**Registration:**

NCT04313621.

**Supplementary Information:**

The online version contains supplementary material available at 10.1007/s40279-025-02214-5.

## Key Points

**Table:** 

The study expands on recent evidence suggesting that Osgood–Schlatter disease is not as benign as previously thought, as it reveals long-term knee issues in adults with a history of the condition.
Significantly lower knee health and a higher risk of developing “jumper’s knee” are seen in adults with a history of Osgood–Schlatter.
These results suggest the need for specific management strategies for patients with Osgood–Schlatter to address the potential long-term impacts of this condition.

## Introduction

Osgood–Schlatter is a common and burdensome apophysitis condition which affects the knee of one in ten adolescents [1–3]. The condition affects the tendon–bone interface at the tibial tubercle during skeletal maturation. The clinical symptoms are pain on palpation, local swelling, and pain during weightbearing physical activity, which causes restricted sports participation and decreased quality of life (QoL) [4–6]. The current understanding of the pathogenesis is that ongoing traction and tensile loading from the major load-bearing patellar tendon on the secondary ossification center make the tendon–bone interface more prone to microavulsions and irritation of the involved tissues [5, 7, 8]. In line with this, symptoms and prevalence seem to be more common among adolescents with a higher level of physical activity [1, 9].

Although Osgood–Schlatter historically has been considered to be a benign and self-limiting condition, studies have emerged on the effects of Osgood–Schlatter 2–8 years from diagnosis [11–14]. These studies have found an increased level of sustained knee pain and decreased function, sports participation, and quality of life [11–14]. The age at follow-up in these studies (mean age ranged from 14 to 21 years) suggests that sequelae persist beyond the expected morphological maturation of the tibial tubercle, but it remains uncertain if this trajectory is continued into later adulthood, as no study has investigated this. Despite the evidence, most clinicians with a special interest in Osgood–Schlatter still consider the condition to be generally benign, believing that for most patients with the condition, it will subside within a year along with the closure of the secondary ossification center [10]. In addition, they often believe that patients will return to pain free participation in physical activity and sports in adulthood [10].

### Objectives

The purpose of this study is to investigate the long-term knee health in adults with a history of Osgood–Schlatter during adolescence. This will be achieved with the following three objectives:

1. To describe the self-reported knee health, prevalence of knee-related comorbidities, and current knee symptoms across different age groups of adults in Denmark with a history of Osgood–Schlatter in their adolescence.
2. To compare the self-reported knee health, prevalence of knee-related comorbidities, and current knee symptoms with estimates from healthy populations sourced from existing literature.
3. To examine if self-reported historical Osgood–Schlatter symptoms (such as duration, symptom severity, and restrictions in participation) are associated with current self-reported knee health and prevalence of knee-related comorbidities by comparing subgroups on the basis of prespecified explanatory variables. We expect adults with a history of Osgood–Schlatter to be affected in the long-term compared with healthy population estimates.

## Methods

### Study Design and Setting

This study is an exploratory population-based cohort study with a cross-sectional follow-up with patient-reported survey data on knee health, knee-related comorbidities, knee symptoms, and historical apophysitis symptoms. Data were collected from patients with a lower-limb apophysitis who were diagnosed and registered in the Danish private or public secondary care in the years 1977–2020. The study was preregistered (ClinicalTrials.gov: NCT04313621, study protocol: 10.1101/2020.03.01.20029660). The study was approved by the regional ethics review board (Committee on Health Research Ethics for The Capitol Region, Denmark, H-20016972) and the Capital Region Data Protection Agency (P-2020-433). The Danish Health Data Authority (FSEID-00005577) approved access to the National Patient Registry (NPR).

### Deviations from Protocol

This paper only reports data for cases with Osgood–Schlatter and knee-related outcomes, although the study also collected data for adults with a history of Sever’s disease (calcaneal apophysitis) and Sinding-Larsen Johansson syndrome (patella-pole apophysitis), as well as other outcomes such as self-rated health, other general comorbidities, and health-related characteristics. For assessing the influence of explanatory variables on numerical outcomes, linear regression was planned [16], but the data did not sufficiently meet the required assumptions of normal distribution. We performed Fisher’s exact test on the basis of contingency tables to calculate odds ratios for the dichotomous variables for comorbidities, rather than logistic regression, as individual participant data from the healthy population estimates sourced in existing literature was not available. Contingency tables produce the exact same odds ratio estimates as logistic regression; therefore, this should not affect the robustness of the analysis or introduce risk of selective analysis strategy. The Knee Injury and Osteoarthritis Outcome Score (KOOS) “QoL” subscale score was not mentioned in the protocol because of oversight but is included as an outcome in analyses. All analyses were performed using R, rather than Stata. In the protocol, it was stated that we potentially would add relevant subgroups should they emerge, and we have thus post hoc added “jumper’s knee” as a specific outcome, as we found a very large prevalence for this comorbidity among cases.

### Participants

Eligible participants had to meet the following criteria: (1) be 18–55 years old, (2) have been diagnosed with Osgood–Schlatter at a Danish private or public hospital between 1977 and 2020, and (3) be a Danish citizen with a social security number. No a priori target sample size was set, as the available sample population was unknown. Besides being < 18 or > 55 years old, we did not impose any exclusion criteria. Patients registered in the NPR with Osgood–Schlatter during adolescence (< 18 years) in relation to a specialized care visit between 1977 and 2020 were subsequently surveyed once in 2021, regardless of their current age.

### Data Collection

Follow-up data collection was performed in April 2021 when the survey was sent to participants, followed by three reminders over the following 4 weeks. Survey invitations were sent to the participant's digital government-issued inbox. Eligible participants were identified using their unique social security number (CPR number), obtained from the NPR. The follow-up duration for participants (time from diagnosis) was 2–46 years. The survey was completed in Research Electronic Data Capture (REDCap) [15], a logged secure system designed for capturing sensitive noncommercial research data. A Danish version of the survey is available in the study protocol [16].

### Comparison with Existing Literature

Several sources from the existing literature were used to extract the knee health estimates on healthy populations that were used for comparing the surveyed Osgood–Schlatter cases. Sources were identified through systematic searches in the MEDLINE and Cochrane CENTRAL databases and included on the basis of sample size. A study of healthy adults in age groups 18–25, 25–35, 35–45, and 45–55 years in the USA reported healthy population estimates for the Knee Injury and Osteoarthritis Outcome Score (KOOS) subscales (“symptoms,” “pain,” “sport/rec,” and “quality of life”) [17]. For knee-specific comorbidities, studies with prevalence data for a single mean age are used for comparisons [18–21]. No healthy population prevalence estimates could be identified for the prespecified comorbidities “runner’s knee,“ “ligament injury,” or “other acute knee injury,” and thus, no comparisons were made for these conditions. An overview with details of the sources from existing literature for healthy populations and their estimates, along with comparisons with the current sample, is provided in the Sect. 3 (Table 2).

### Descriptive, Explanatory, and Outcome Variables

The following outcomes are arranged from most to least important concerning the study objectives, but they are otherwise exploratory.

#### Knee-Related Health

Participants rated their knee health on the KOOS. The KOOS is composed of five subscales (symptoms, pain, ADL, sport/rec, and quality of life), which is scored independently on a 0–100 scale, with 0 being the worst possible score and 100 being the best possible score. The ADL subscale was omitted owing to low responsiveness in non-osteoarthritic and non-surgical populations. Participants also rated their worst knee pain during the past month on a 0–100 numerical pain rating scale (NPRS), as well as current knee pain/problems in the same area (as the Osgood–Schlatter) on a 4-point Likert scale (“yes, to a severe extent,” “yes, to some extent,” “yes, to a lesser extent,” or “no, not at all’), and whether they had a sustained bony prominence at the tibial tubercle (“no,” “small,” or “large”).

#### Knee-Specific Comorbidities

Participants were asked if they have had any of five prespecified knee-specific comorbidities; jumper’s knee (“yes” or “no”), runner’s knee (“yes” or “no”), anterior cruciate ligament injury (“yes” or “no”), other ligament injury (“yes” or “no”), or meniscal injury (“yes” or “no”).

#### Explanatory and Descriptive Variables

Explanatory variables were duration of Osgood–Schlatter (< 1 month, 1–3 months, 3–6 months, 6–12 months, 1–2 years, 2–4 years, > 4 years, or “don’t know/can’t recall”), pain level during Osgood–Schlatter (“a lot of pain,” “some pain,” “little pain,” “no pain, other symptoms,” or “don’t know/can’t recall”), level of restrictions during Osgood–Schlatter (“no limitations,” “small limitation,” “some limitations,” “very limited,” “total limitation,” or “don’t know/can’t recall”), and current level of bony prominence of the tibial tubercle (“no prominence,” “small prominence,” “large prominence,” or “don’t know”). Demographic variables were age (years), sex (female/male), and body mass index (kg/m^2^).

### Subgrouping

The major source in the existing literature of healthy population estimates for KOOS scores divides participants into subgroups on the basis of age groups (18–25, 25–35, 35–45, and 45–55 years) and sex [17, 22]. For available individual comorbidity prevalence estimates, we used the mean age provided to select a corresponding age group for comparison. For example, for anterior cruciate ligament injury, the mean age for the prevalence estimate was 28 years, and this was then compared solely to the cases/noncases reported in the 25–35 year age group. For descriptive and comparative analysis, we maintained these eight sex/age groups.

### Statistical Methods

Missing data are reported in tables for each variable but has been removed from all analysis with no imputations. Outliers with an impossible combination of answers were excluded. For contingency tables without cases, data were omitted from plots, and no estimates were produced. “Don’t know”/“don’t recall”-type responses have been omitted as categories before analyses but are denoted in tables. For the association analyses, the prespecified strata of age and sex were omitted but included in descriptive plots. All analyses for all variables are denoted in Supplementary Table S1 to provide an overview of which tests were performed.

For descriptive data, no statistical analysis was performed, but they are reported in text and plots using counts, means, or medians, with measures of variance (95% confidence intervals or interquartile ranges).

For comparing Osgood–Schlatter cases with healthy population estimates of KOOS scores, we used the unpaired Welch *t*-test. For comparing comorbidity prevalence for Osgood–Schlatter cases with population prevalence, we calculated the odds ratios and 95% confidence intervals from contingency tables and used Fisher’s exact test for significance tests.

For investigating associations between explanatory variables and outcomes, we used a stepwise approach. We first used the nonparametric Kruskal–Wallis test between groups (e.g., between different durations of Osgood–Schlatter). If Kruskal–Wallis was significant, the pairwise Wilcoxon sign rank test was used, with a Bonferroni–Holm correction for multiple comparisons to mitigate family-wise error rate across explanatory outcome matrices. For determining association between categorical explanatory variables and categorical outcomes, we calculated *p* values from the Spearman rank correlation test only if the Kruskal–Wallis test was significant when comparing the explanatory variable to the different outcome categories.

Between-group differences are denoted using a numerical scale or standardized effect sizes (Cohen’s *d*) assessed as trivial (*d* < 0.2), small (*d* ≥ 0.2), medium (*d* ≥ 0.5), and large (*d* ≥ 0.8), as appropriate. The denoted magnitude of odds ratio is based on a range of confidence intervals, from five predefined levels of magnitude ranging on the basis of GRADE definitions of magnitudes. These range from “very large difference" in risk (OR < 0.2, or > 5.0) to “negligible difference” (OR 0.91–1.20) on the basis of GRADE definitions of magnitudes [16, 23]. All analyses were performed in R using the RStudio software (R 4.3.2, Foundation for Statistical Computing, Vienna, Austria; RStudio 2023.06.0).

## Results

### Flow of Participants

In the Danish National Patient Registry, we identified 1218 eligible adults who received a diagnosis of Osgood–Schlatter during the period 1977–2020 and were invited to participate in this study. Of these, 400 (33%) responded to the survey. In total, 3 respondents were excluded owing to improbable combinations of answers, and thus, 397 participants were included in the analyses.

### Descriptive Results

The descriptive results are presented in Table 1 (knee-related health) and Table 2 (comorbidities). More than half (*n* = 204, 51%) of the participants reported their duration of Osgood–Schlatter symptoms as lasting ≥ 2 years (“don’t recall duration”: *n* = 91, 23%). In terms of limitation in sports participation, 47% (*n* = 188) responded they were “very” or “totally” limited during their Osgood–Schlatter. Sustained bony prominence was reported by 85% (*n* = 339).

**Table 1:** Descriptive results for participant characteristics, historical and current symptoms, and KOOS subscale scores

Variables/age group	18–55 years	(*n* = 397)	18–25 y	(*n* = 153)	25–35 years	(*n* = 74)	35–45 years	(*n* = 44)	45–55 years	(*n* = 126)
	%	*n*	%	*n*	%	*n*	%	*n*	%	*n*
Sex (% female)	35	*n* = 139	32	*n* = 49	31	*n* = 42	25	*n* = 11	38	*n* = 48
Overweight (BMI > 25 kg/m^2^)	50	*n* = 200	31	*n* = 47	45	*n* = 33	61	*n* = 27	74	*n* = 93
*Current pain/problem from same area*										
Not at all	27	*n* = 106	25	*n* = 36	22	*n* = 16	20	*n* = 9	36	*n* = 45
To a small degree	38	*n* = 149	39	*n* = 60	36	*n* = 27	39	*n* = 17	36	*n* = 45
To some degree	27	*n* = 109	28	*n* = 43	34	*n* = 25	30	*n* = 13	22	*n* = 28
To a severe degree	8	*n* = 33	9	*n* = 14	8	*n* = 6	11	*n* = 5	6	*n* = 8
*Sustained bony prominence*										
Don’t know	8	*n* = 30	8	*n* = 13	3	*n* = 2	7	*n* = 3	9	*n* = 11
No prominence	7	*n* = 28	8	*n* = 13	8	*n* = 6	5	*n* = 2	6	*n* = 7
Small prominence	33	*n* = 132	39	*n* = 59	32	*n* = 23	25	*n* = 11	31	*n* = 39
Large prominence	53	*n* = 207	44	*n* = 68	58	*n* = 42	64	*n* = 28	51	*n* = 69
*Recalled duration of apophysitis*										
Do not know/cannot recall	23	*n* = 91	14	*n* = 21	18	*n* = 13	23	*n* = 10	37	*n* = 46
< 1 month	0	*n* = 0	0	*n* = 0	0	*n* = 0	0	*n* = 0	0	*n* = 0
1–3 months	1	*n* = 5	2	*n* = 3	0	*n* = 0	0	*n* = 0	2	*n* = 2
3–6 months	3	*n* = 12	3	*n* = 4	4	*n* = 3	2	*n* = 1	3	*n* = 4
6–12 months	8	*n* = 30	12	*n* = 19	5	*n* = 4	0	*n* = 0	5	*n* = 7
1–2 years	14	*n* = 55	17	*n* = 26	12	*n* = 9	14	*n* = 6	11	*n* = 14
2–4 years	15	*n* = 60	18	*n* = 27	16	*n* = 12	11	*n* = 5	12	*n* = 16
> 4 years	36	*n* = 144	35	*n* = 53	45	*n* = 32	50	*n* = 22	30	*n* = 37
*Recalled pain during apophysitis*										
Do not know/cannot recall	3	*n* = 11	2	*n* = 5	0	*n* = 1	0	*n* = 0	4	*n* = 5
No pain, other symptoms	1	*n* = 4	1	*n* = 2	0	*n* = 0	0	*n* = 0	16	*n* = 2
Little pain	12	*n* = 48	8	*n* = 12	11	*n* = 8	11	*n* = 5	18	*n* = 23
Some pain	50	*n* = 201	48	*n* = 74	48	*n* = 35	75	*n* = 33	47	*n* = 59
A lot of pain	34	*n* = 133	39	*n* = 60	41	*n* = 30	14	*n* = 6	29	*n* = 37
*Recalled participation limitations due to apophysitis*										
Do not know/cannot recall	1	*n* = 5	1	*n* = 2	0	*n* = 1	2	*n* = 0	2	*n* = 2
No limitation	2	*n* = 8	1	*n* = 2	1	*n* = 1	2	*n* = 1	3	*n* = 4
Small limitation	15	*n* = 58	12	*n* = 18	16	*n* = 12	14	*n* = 6	17	*n* = 22
Some limitations	35	*n* = 138	29	*n* = 45	40	*n* = 29	36	*n* = 16	38	*n* = 48
Very limited	32	*n* = 127	37	*n* = 57	27	*n* = 20	41	*n* = 18	25	*n* = 32
Total limitation	15	*n* = 61	19	*n* = 29	15	*n* = 11	7	*n* = 3	14	*n* = 18
*Mean KOOS subscale scores (0–100)*	Points	[95% CI]	Points	[95% CI]	Points	[95% CI]	Points	[95% CI]	Points	[95% CI]
Pain	83.2	[81–85]	81.3	[80–86]	84.4	[84–88]	83.9	[79–89]	82.2	[79–85]
Symptoms	82.4	[81–84]	82.9	[80–85]	84.5	[81–88]	81.1	[75–87]	81.0	[78–84]
Sport/Rec	64.9	[62–68]	66.8	[63–71]	64.8	[59–71]	67.0	[60–74]	62.0	[57–67]
QoL	66.8	[64–69]	68.0	[64–72]	65.7	[60–71]	65.1	[57–73]	66.7	[62–71]
	Median	IQR	Median	IQR	Median	IQR	Median	IQR	Median	IQR
*Worst knee pain past month (0–100 NPRS)*	15	5–50	15	5–45	15	5–30	20	5–60	15	8–50

*KOOS* Knee Injury and Osteoarthritis Outcome Score, *Rec* recreation function, *QoL* quality of life, *NPRS* numerical pain rating scale, with 0 being “no pain” and 100 being “worst pain imaginable,” *IQR* interquartile range, *BMI* body mass index

**Table 2:** Prevalence estimates of knee-specific comorbidities from healthy populations and Osgood–Schlatter cases

	Osgood–Schlatter cases					Healthy populations							Risk differences	
Condition	Comparator group	*n*	Cases (*n*)	Noncases (*n*)	Proportion of cases (%)	Age	Sex distribution	*n*	Cases (*n*)	Noncases (*n*)	Proportion of cases (%)	Country, setting	Odds ratio 95% CI	*p*-Value
Meniscal injury	Female, 18–25 years	49	2	47	4.1	16–19 years	100% females	361,707	1000	360,707	0.3	Israel, medical evaluation for mandatory military service	3.7–63.5	*p* = 0.0082
Meniscal injury	Male, 18–25 years	104	4	100	3.8	16–19 years	100% males	463,480	2806	460,674	0.6	Israel, medical evaluation for mandatory military service	2.4–18.0	*p* = 0.0037
Knee osteoarthritis	Female, 18–25 years	49	0	49	0.0	16–24 years	100% females	9202	46	9156	0.5	Denmark, general population survey (self-reported)	–	–
Knee osteoarthritis	Male, 18–25 years	104	4	100	3.8	16–24 years	100% males	6997	35	6962	0.5	Denmark, general population survey (self-reported)	2.7–22.8	*p* = 0.0024
Knee osteoarthritis	Female, 25–35 years	31	0	31	0.0	25–34 years	100% females	8016	216	7800	2.8	Denmark, general population survey (self-reported)	–	–
Knee osteoarthritis	Male, 25–35 years	43	2	41	4.7	25–34 years	100% males	6086	164	5922	2.8	Denmark, general population survey (self-reported)	0.4–7.3	*p* = 0.3255
Knee osteoarthritis	Female, 35–45 years	11	0	11	0.0	35–44 years	100% females	12,340	1333	11,007	12.1	Denmark, general population survey (self-reported)	–	–
Knee osteoarthritis	Male, 35–45 years	33	5	28	15.2	35–44 years	100% males	9774	1056	8718	12.1	Denmark, general population survey (self-reported)	0.5–3.8	*p* = 0.3966
Knee osteoarthritis	Female, 45–55 years	48	12	36	25.0	45–54 years	100% females	16,031	5162	10,869	32.2	Denmark, general population survey (self-reported)	0.3–1.3	*p* = 0.3536
Knee osteoarthritis	Male, 45–55 years	78	15	63	19.2	45–54 years	100% males	13,533	4358	9175	32.2	Denmark, general population survey (self-reported)	0.3–0.9	*p* = 0.1458
Jumper’s knee	Male, 25–35 years Female, 25–35 years	74	31	43	41.9	Mean 32.5 ± 10.7 years	50% females	1000	10*	990	1.1*	Denmark, consecutive patients in general practice	32.9–155.0	*p* < 0.0001
ACL injury	Male, 25–35 years	43	1	42	2.3	Mean 27.9 ± 6.8 years	98% males	320	47	273	14.7	Saudi Arabia, soccer players	0.03–0.8	*p* = 0.0276

*ACL* anterior cruciate ligament

*Age and sex from entire sample not available. Cases were disturbed equally across sex (five females and five males)

### KOOS

**Fig. 1:**
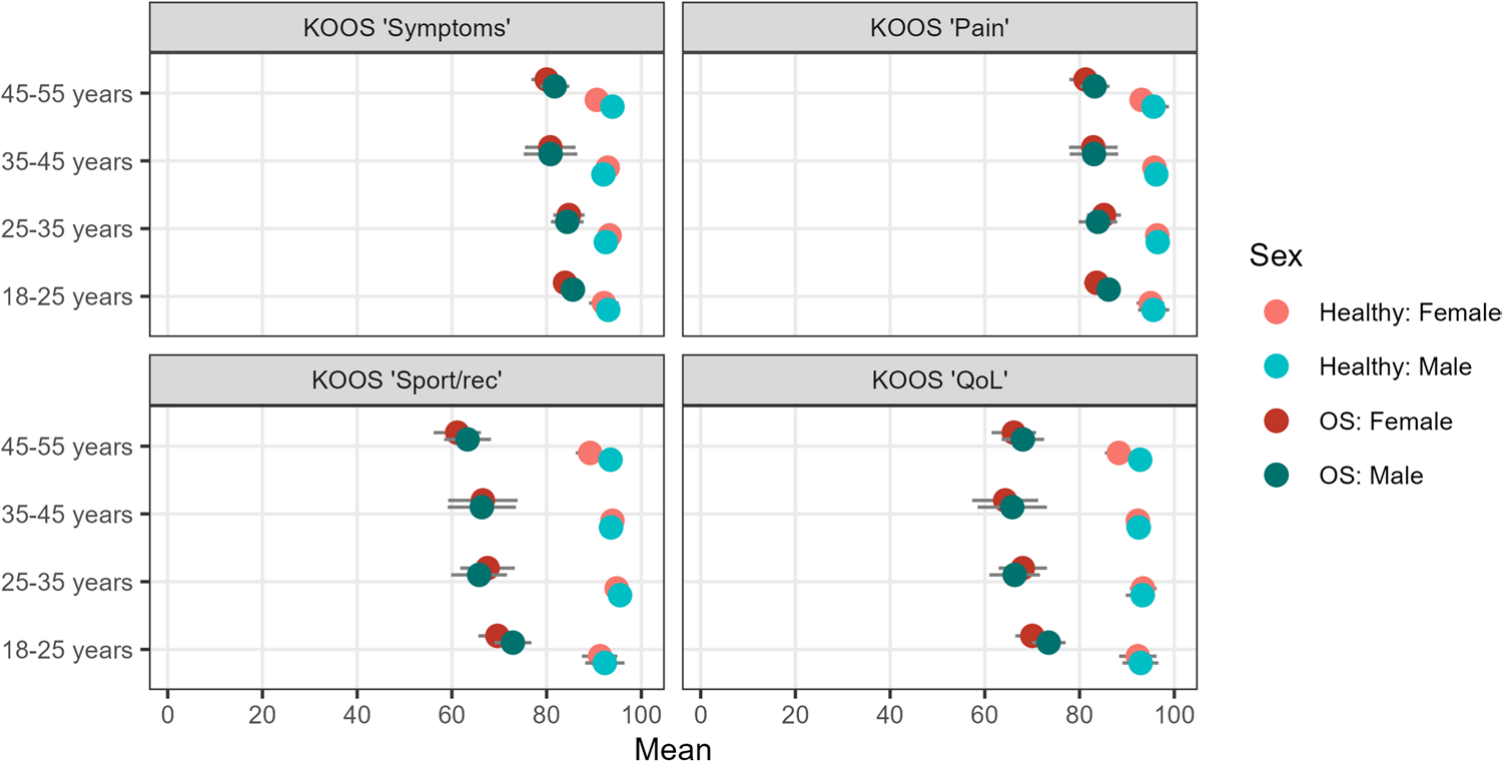
Self-reported knee health on KOOS subscales compared with healthy population estimates; *OS* Osgood–Schlatter, *KOOS* Knee Injury and Osteoarthritis Outcome Score, *Rec* recreation function, *QoL* quality of life

For KOOS subscales “sport/rec” and “quality of life,” across all age/sex groups, the Osgood–Schlatter cases scored significantly lower than the healthy population estimates (between-group differences ranged from 19 to 38 points, Cohen’s *d* = 0.83–1.65, *p* = < 0.001 to 0.019, see Fig. 1). Except for females aged 35–45 years (*n* = 11 with Osgood–Schlatter, between-group differences ranged from 5.6 to 12.3 points, Cohen’s *d* = 0.5–0.6, *p* = 0.073–0.094), the same was observed for subscales “symptoms” and “pain” (*p* = 0.003 to < 0.001, between-group difference 7–19 points, Cohen’s *d* = 0.5–1.2).

### Knee-Related Comorbidities

**Fig. 2:**
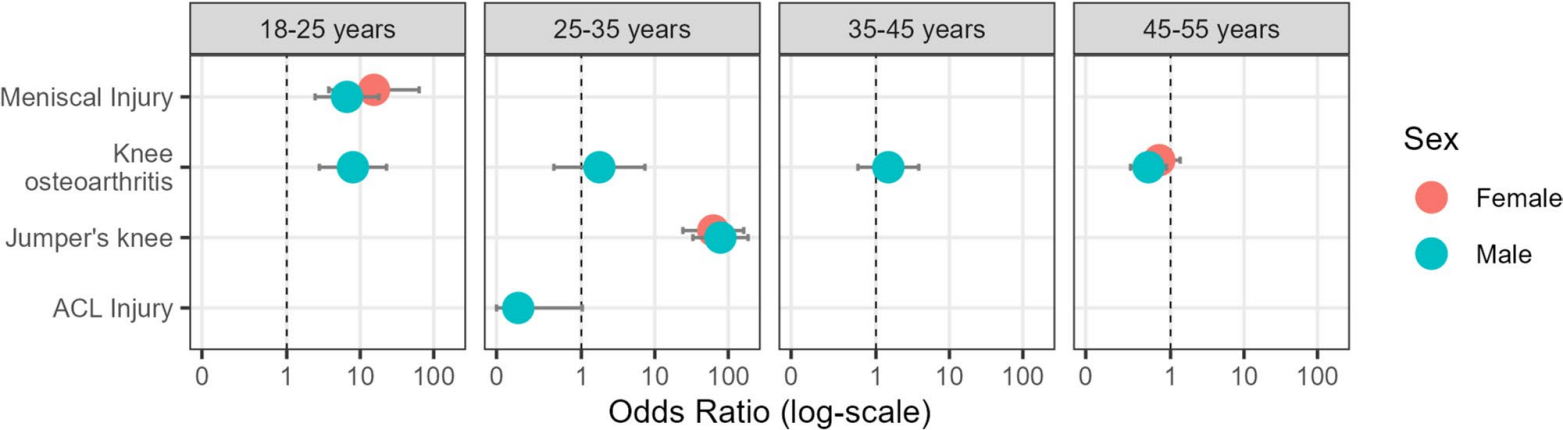
Odds of having knee-related comorbidities compared with estimates from healthy populations; *ACL* anterior cruciate ligament

There was a very large risk for jumper’s knee (odds ratio: 70.4 [95% CI 32.9, 155.0], *p* < 0.001, see Fig. 2 and Table 2), and large-to-very-large risk for meniscal injury (odds ratio: 15.3 [95% CI 3.7, 63.3], *p* = 0.008 for women; and odds ratio: 6.6 [95% CI 2.4, 17.9], *p* = 0.004 for men, see Fig. 2 and Table 2) for Osgood–Schlatter cases. For anterior cruciate ligament injuries, a small-to-very-large risk was observed (odds ratio: 0.1 [95% CI 0.003, 0.8], *p* = 0.028). For knee osteoarthritis, a moderate-to-very-large risk was observed for males 18–25 years old (odds ratio: 7.9 [95% CI 2.0, 22.8], *p* = 0.002), and conversely, a small-to-large reduced risk among males aged 45–55 years (odds ratio: 0.5 [95% CI 0.3, 0.9], *p* = 0.014) among Osgood–Schlatter cases.

### Association of Self-Reported Historical Apophysitis Symptoms and Outcomes

#### Duration of Symptoms

**Fig. 3:**
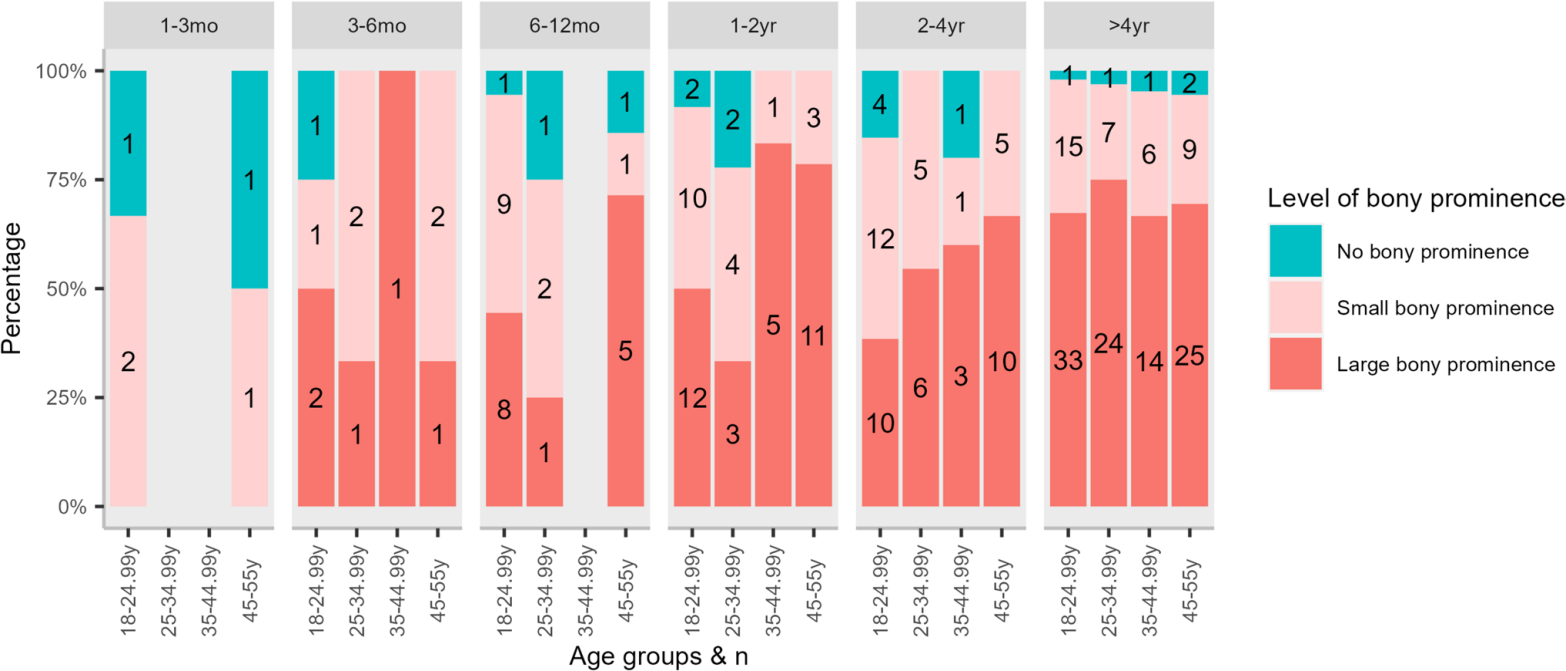
Distribution of level of tibial bony prominence, by duration of Osgood–Schlatter. Figures in bars denotes the number of participants in for each subgroup

**Fig. 4:**
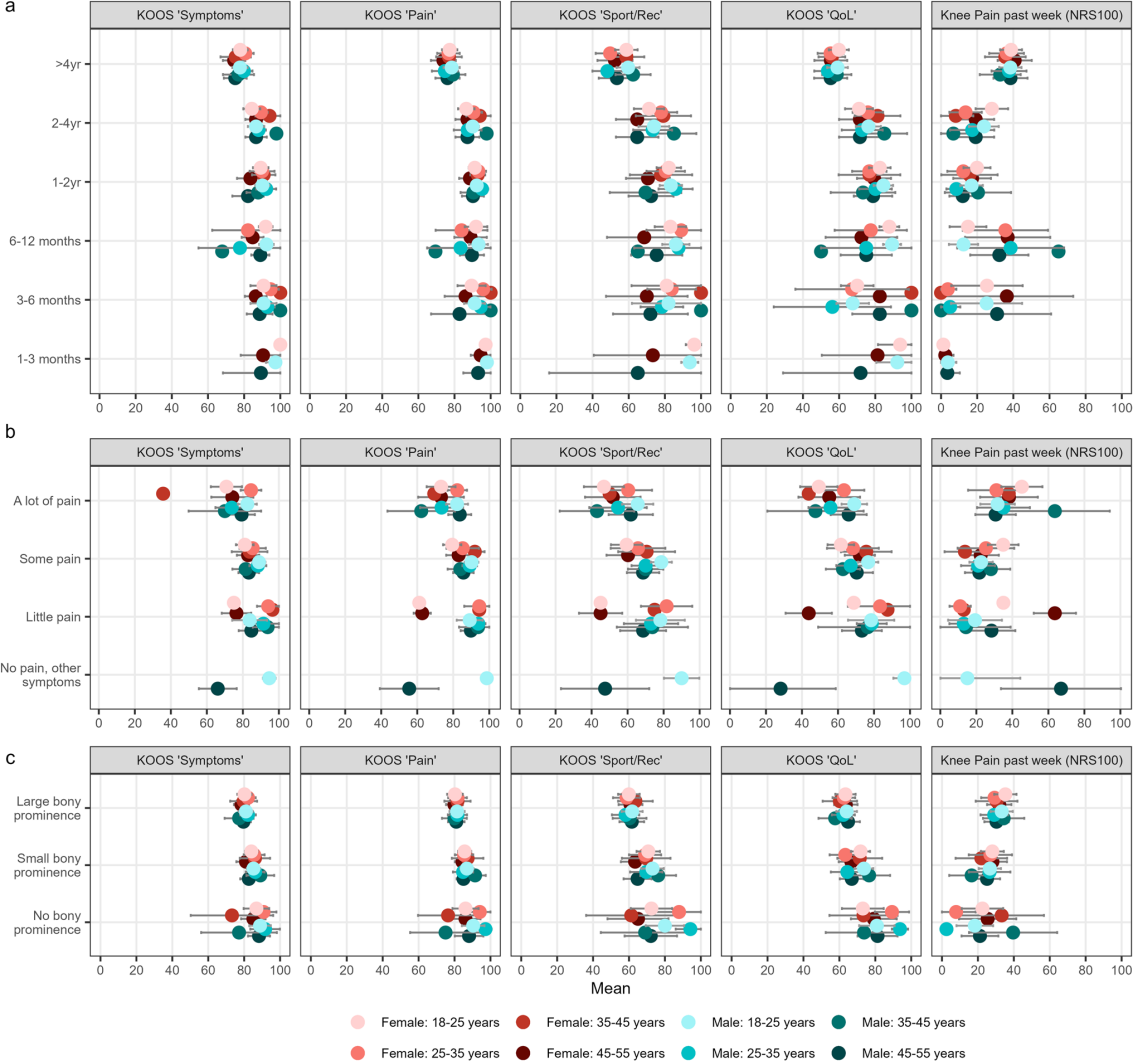
Distribution of self-reported numerical outcomes by Osgood–Schlatter duration (**a**), pain during Osgood–Schlatter (**b**), and presence of current bony prominence at the tibial tubercle (**c**). Error bars denote 95% confidence intervals. KOOS subscales are scored from 0 to 100, with 0 representing extreme knee problems and 100 representing no knee problems. Knee pain is scored on a 0–100 numerical rating scale with zero representing no knee pain and 100 representing worst pain imaginable. *KOOS* Knee Injury and Osteoarthritis Outcome Score, *Rec* recreation function, *QoL* quality of life

Increased duration of symptoms was associated with the presence of a bony prominence (*p* < 0.001) (Fig. 3). Participants who reported a symptom duration of > 4 years reported significantly decreased KOOS “symptoms,” “pain,” and “sport/rec” scores (“symptoms” *p*-value range: 0.004–0.03, “pain” *p*-value range: *p* = 0.009 to < 0.001, and “sport/rec” *p*-value range: *p* = 0.017 to < 0.001) compared with all the subgroups of participants with other durations of symptoms, except for those who reported symptoms for 1–3 months (*n* = 5, “symptoms”: *p* = 0.083, “pain”: *p* = 0.067, “sport/rec”: *p* = 0.154) (Fig. 4a). Similarly, KOOS subscale “QoL” scores were significantly lower for participants reporting a duration of > 4 years compared with durations of 6–12 months (*n* = 30), 1–2 years (*n* = 55), and 2–4 years (*n* = 60) (*p* < 0.001), but not compared with durations of 1–3 months (*p* = 0.150, *n* = 5) and 3–6 months (*p* = 0.110, *n* = 12). Also, the participants with a symptom duration of >4 years reported significantly higher current knee pain compared with all the other subgroups (*p* value range: 0.018 to < 0.001). The duration of symptoms did not affect the prevalence of jumper’s knee (*p* = 0.606).

#### Pain Level During Osgood–Schlatter

For current knee pain and on all four KOOS subscales, reporting “a lot of pain” during Osgood–Schlatter lead to significantly worse scores than reporting “some pain” (*p* < 0.021) (Fig. 4b). No other differences were observed between other recalled pain levels on either KOOS subscales or knee pain (*p* > 0.05). Difference in recalled pain levels did not affect the prevalence of jumper’s knee (*p* = 0.756) or the presence of a bony prominence (*p* = 0.252).

#### Limitations on Sports and Physical Activity During Osgood–Schlatter

There were no significant differences in koos subscales, current knee pain, prevalence of jumper’s knee, or the presence of a bony prominence between subgroups on the basis of levels of limitations during Osgood–Schlatter (*p*-value range = 0.344–0.057).

#### Current Bony Prominence

Having a large versus no bony prominence resulted in worse KOOS “pain” scores (*p* = 0.034) (Fig. 4c). For KOOS “sport/rec,” a large bony prominence resulted in worse scores compared with both none and small (*p* = 0.016). The same was observed for the KOOS "QoL" (*p* = 0.002 and *p* = 0.042, respectively). In addition, small versus no bony prominence also showed lower KOOS “QoL” scores (*p* = 0.012). Current knee pain was worse for those reporting large versus no bony prominence (*p* = 0.023). Having large, small, or no bony prominence did not result in changes in jumper’s knee prevalence (*p* = 0.057).

## Discussion

### Key Results

The purpose of this study was to investigate long-term knee health in adults with a history of Osgood–Schlatter. We observed moderate-to-large decreases in self-reported KOOS subscale scores for Osgood–Schlatter cases compared with healthy population estimates across all age groups, up to 55 years of age. In addition, we observed a very large prevalence of jumper’s knee, and large risk for meniscal injury. More than two-thirds had current pain or problems from the same area around the tibial tubercle, and more than half had a large sustained bony prominence of the tibial tubercle. Most still had knee pain during the previous month (84%). An increased duration of symptoms and pain level during Osgood–Schlatter was associated with outcomes.

### Interpretations and Generalizability

The findings of the present study are in line with five previous studies, across different settings, with smaller samples and shorter follow-up. The previous studies found decreased KOOS scores [12, 13], sustained knee pain [12, 14], altered morphology [11, 14], decreased sports participation [7, 12, 14], and impaired clinical strength and endurance among patients with a history of Osgood–Schlatter [11]. However, the current findings contradict established textbook knowledge, surveys of clinicians, and narrative reviews that characterise the condition as innocuous and with a good prognosis, restricted to the years around the growth spurt [5, 8, 24–26]. On the contrary, we observed detrimental outcomes across all age groups, into late adulthood.

Our findings indicate that Osgood–Schlatter might have the potential to affect knee health in the long-term. The combined findings among the Osgood–Schlatter cases of sustained local pain, bony prominence, high jumper’s knee prevalence, and decreased KOOS scores might point to sustained changes or pathology in the involved tissues that persist into adulthood. In the following sentences, we hypothesize how these findings could relate to some of the characteristics of adolescent Osgood–Schlatter. First, the presence of a free ossicle anterior to the secondary ossification center of the tibial tubercle [27] might lead to incomplete fusion of the apophysis junction. This incomplete fusion might lead to local suboptimal transfer of tensile load at the tendon attachment point on fragmented or irregular bone, potentially causing overuse of the adjacent tendon tissue, which could persist as tendon-related complaints. If there is a persistent ossicle or abnormal calcification elsewhere on the apophysis, this may also result in shortening of the patellar tendon, leading to suboptimal biomechanical transfer of tensile load. Second, there is a potential local response of increased neovascularization, fluid retention, and fibrillary disorganization which has the potential to persist [28, 29]. Along with the abnormal attachment, this combination may predispose the tendon to tendinopathy symptoms or lead to tendinosis changes in adulthood. In clinical practice, we have anecdotally observed that palpation pain and morphological changes in the tendon can persist even after the load-related pain and pain on palpation of the tibial tubercle have subsided and full maturation of the apophysis has occurred.

Adolescents with severe Osgood–Schlatter often exhibit a history of high levels of sports participation and a strong desire to return to their previous activity levels. Continuous attempts to return to the same level may exacerbate some of these maladaptive responses, further elevating the risk of adverse long-term outcomes. There might be some inherent morphological differences between adolescents who develop Osgood–Schlatter and those who do not, and these differences might also be responsible for the increased risk of adult patella tendinopathy, rather than being two conditions on the same spectrum.

### Study Limitations and Strengths

Our study has methodological limitations that limit the extrapolation of our findings to the target population. While the Danish national patient registry provides the most exhaustive source of data in Denmark when studying a specific patient population, the registry only includes patients with a CPR number, and no other residents such as exchange students, immigrant workers, marginalized people without documentation, and so on. Furthermore, there is an error rate of 0–2% and a risk of mislabeling bias and missed cases due to misclassification, as the data in the registry are produced by health care professionals during daily operations/tasks. Owing to the nature of convenience sampling from exhaustive registries, we did not perform a priori power calculations to determine the sample size needed, but the width of the confidence intervals does not suggest issues with statistical power. The data have a risk of spectrum bias, as more severe cases are more likely to be included in the source population compared with the target population, owing to data being drawn from secondary care, which represents patients seeking specialized treatment. This risk is also present at the study population level, as those who chose to respond (33 %) may have been more likely to prioritize survey participation if they had persistent problems or experienced a burdensome disease trajectory during adolescence. This means that the majority of potential participants, the non-responders (66%), could represent a less severe subgroup of patients, which may have inflated the estimates in our sample compared with the true estimates. Differences observed between Osgood–Schlatter cases and the healthy population estimates could be inflated as a result of cases potentially being more exposed to high-load physical activities and sports. Nevertheless, these potentially severe cases are also the patients that are most in need of increased care to mitigate and manage potential long-term effects. The KOOS estimates for a healthy population used the English version of KOOS, which might introduce some bias owing to the lack of cross-cultural validation [30]. The multiple measures, comparisons, and analyses required conservative corrections of the *p* values, but the risk of false positive findings is present and should be included in the interpretation of the certainty and magnitude of the results. The study had no control over enrollment in the cohort, as the Osgood–Schlatter cases were extracted from the national patient registry. The study is at risk of recall bias as we only have a single follow-up timepoint, which ranged from 2 to 46 years from diagnosis.

## Conclusions

Adults with a history of Osgood–Schlatter during adolescence have significantly decreased KOOS scores when compared with estimates from a healthy population. Sustained bony prominence and symptoms from this area were present in three out of four adults. Furthermore, a large prevalence of “jumper’s knee” was present compared with estimates from healthy populations. Long-term duration of symptoms and high pain levels when having Osgood–Schlatter were negatively associated with KOOS outcomes. These findings suggest that Osgood–Schlatter may not be as benign and self-limiting as traditionally believed. This perspective is supported by other recent studies showing persistent symptoms and prolonged trajectories, challenging the long-held assumptions about the natural course of the condition. This indicates that advice and information to patients with Osgood–Schlatter should address the negative association seen between long duration and high pain levels in adolescence and associated poor long term knee health. Additionally, this information should potentially guide management strategies to maintain knee health over time.

## Supplementary Information

Below is the link to the electronic supplementary material.

Supplementary file1 (DOCX 15 KB)

## Declarations

### Data availability statement

Anonymous data with anthropometrics and prose-responses removed are available at the following repository, along with scripts used for data cleaning and analyses: 10.6084/m9.figshare.28581110.
