# Supplementary material for: Long-Term Knee Health in Adults with a History of Adolescent Osgood–Schlatter: A National Cohort Study of Patients in Secondary Care in Denmark 1977–2020
Source: Sports Med. 2025 May 29;55(7):1769–81. doi: 10.1007/s40279-025-02214-5 (PMC12296839; doi:10.1007/s40279-025-02214-5)
Supplement: Supplementary file 1 — Supplementary file1 (DOCX 15 KB) [file 40279_2025_2214_MOESM1_ESM.docx]

| **Table S1. Statistical comparisons, associations, and test used for all variables** | | |
| --- | --- | --- |
| **Differences between cases and healthy population** | |  |
| **Analysis** | **Outcome variables** |  |
| Unpaired welch t-test | KOOS subscales scores (0-100) |  |
| Unpaired welch t-test | Worst knee pain past month (0-100) |  |
| Fishers exact test | Jumpers knee (Y/N) |  |
| Fishers exact test | ACL injury(Y/N) |  |
| Fishers exact test | Meniscal injury (Y/N) |  |
| Fishers exact test | Knee osteoarthritis (Y/N) |  |
| **Associations of explanatory variables with outcome variables** | | |
| **Analysis** | **Outcome variables** | **Explanatory variables** |
| Kruskal Wallis, if significant then Wilcoxon Sign Rank | KOOS subscales scores (0-100) | Current pain/problem from same area (1-4).  Sustained bony prominence (1-3)  Recalled duration of apophysitis (1-7)  Recalled pain during apophysitis (1-5) Recalled participation limitations due to apophysitis (1-5) |
| Kruskal Wallis, if significant then Wilcoxon Sign Rank | Worst knee pain past month (0-100) | Current pain/problem from same area (1-4).  Sustained bony prominence (1-3)  Recalled duration of apophysitis (1-7)  Recalled pain during apophysitis (1-5) Recalled participation limitations due to apophysitis (1-5) |
| Kruskal Wallis, if significant then Spearmans Rank | Jumpers knee (Y/N) | Current pain/problem from same area (1-4).  Sustained bony prominence (1-3)  Recalled duration of apophysitis (1-7)  Recalled pain during apophysitis (1-5) Recalled participation limitations due to apophysitis (1-5) |
| Kruskal Wallis, if significant then Spearmans Rank | Current pain/problem from same area (1-4) | Sustained bony prominence (1-3)  Recalled duration of apophysitis (1-7)  Recalled pain during apophysitis (1-5) Recalled participation limitations due to apophysitis (1-5) |
| Kruskal Wallis, if significant then Spearmans Rank | Sustained bony prominence (1-3) | Current pain/problem from same area (1-4).  Recalled duration of apophysitis (1-7)  Recalled pain during apophysitis (1-5) Recalled participation limitations due to apophysitis (1-5) |
| KOOS = Knee injury and Osteoarthritis Outcome Score, ACL = anterior cruciate ligament | | |
